# Role of Portal Vein Embolization in Hepatocellular Carcinoma Management and Its Effect on Recurrence: A Case-control Study

**DOI:** 10.1007/s00268-012-1522-3

**Published:** 2012-03-13

**Authors:** Rohan C. Siriwardana, Chung Mau Lo, See Ching Chan, Sheung Tat Fan

**Affiliations:** 1 Department of Surgery, The University of Hong Kong, 102 Pokfulam Road, Hong Kong, People’s Republic of China; 2 State Key Laboratory for Liver Research, The University of Hong Kong, 102 Pokfulam Road, Hong Kong, People’s Republic of China

## Abstract

**Background:**

Liver regeneration that occurs after portal vein embolization (PVE) may have adverse effects on the microscopic tumor foci in the residual liver mass in patients with hepatocellular carcinoma (HCC).

**Methods:**

Fifty-four HCC patients with inadequate functional residual liver volume were offered PVE during a seven-year period. Among them, 34 (63%) patients underwent curative resection. They were compared with a matched control group (*n* = 102) who underwent surgery without PVE. Postoperative complications, pattern of recurrence, and survival were compared between groups.

**Results:**

In the PVE group, a pre-embolization functional residual liver volume of 23% (12–33.5%) improved to 34% (20–54%) (*p* = 0.005) at the time of surgery. When the two groups were compared, minor (PVE, 24%; control, 29%; *p* = 0.651) and major (PVE, 18%; control, 15%; *p* = 0.784) complications were similar. After a follow-up period of 35 months (standard deviation 25 months), extrahepatic recurrences were detected in 10 PVE patients (29%) and 41 control patients (40%) (*p* = 0.310). Intrahepatic recurrences were seen in 10 (29%) and 47 (46%) cases (*p* = 0.109) in the PVE and control groups, respectively. In the PVE group, 41% (*n* = 14) of the recurrences were detected before one year, compared with 42% (*n* = 43) in the control group (*p* = 1). Disease-free survival rates at 1, 3, and 5 years were 57, 29, and 26% in the control group and 60, 42, and 42% in the PVE group (log-rank, *p* = 0.335). On multivariate analysis, PVE was not a factor affecting survival (*p* = 0.821).

**Conclusions:**

Portal vein embolization increases the resectability of initially unresectable HCC due to inadequate functional residual liver volume, and it has no deleterious oncological effect after major resection of HCC.

## Introduction

Surgery is the treatment of choice for patients with hepatocellular carcinoma (HCC) [1]. Yet not all detected HCCs are amenable to surgical resection although the resection rate varies from center to center. A functional residual liver volume (FRLV) in excess of 25% in a normal liver or 40% in a diseased liver is considered necessary to avoid postoperative liver failure [2]. For patients with marginal FRLV, portal vein embolization (PVE) increases the resectability of tumor by a corresponding increase of the contralateral side.

Portal vein embolization for patients with HCC raises special considerations. The effectiveness and safety of the procedure in the presence of background diseased liver and progression of the primary tumor are major concerns. Furthermore, PVE and subsequent resection may promote microscopic tumor foci. Some consider that the same growth factors are accountable for hepatic regeneration following PVE, but others contradict this possibility and suggest that different mechanisms may be involved [3, 4]. Wakabayashi [5], for instance, has reported an increase in extrahepatic tumor recurrence following PVE, while Tanaka et al. [6] has documented increased overall survival in a group of 33 patients. The present study evaluated the short-term and long-term outcomes of resection of HCC in patients who had preoperative PVE against a matched control group of patients who did not have preoperative PVE.

## Patients and methods

The study included 54 consecutive HCC patients with inadequate FRLV who had undergone PVE during the period 2002–2009. Eleven (20%) of them underwent percutaneous PVE, whereas the others (80%) were treated with an open procedure via the ileocolic vein. The patients were reassessed after an interval of 4–6 weeks in terms of liver function, tumor progression, and improvement in liver volume. Resection was considered feasible for patients with FRLV more than 20% of a normal liver, 30% of a fibrotic liver, and 40% of a cirrhotic liver. Patients with liver function test results comparable to pre-embolization results and those who had no evidence of disease progression also proceeded to surgery. Transarterial chemoembolization (TACE) was performed after an interval of two weeks in cases of larger tumors or when additional time was considered necessary for liver regeneration.

Resection was considered not to be feasible for 20 patients. They were offered other forms of therapy on an individual basis. These included radiofrequency ablation, high-intensity focused ultrasound ablation, and repeated cycles of TACE. One patient underwent liver transplantation outside our center. The median overall survival in this group was 10 months (range: 8–11 months). Overall, 34 (63%) patients who had improved FRLV underwent surgery after a median interval of 45 days (range: 26–96 days).

Thirty-four patients (the PVE group) who had undergone resection were compared with a matched control sample. The controls were selected by screening a database of patients who underwent surgery as the first treatment. For each case in the PVE group, three controls were selected, matching the year of surgery, type of resection [7] (grouped according to Brisbane 2000 terminology of liver anatomy and resections), and the presence of vascular permeation.

Postoperative complications in the two groups were categorized according to the Clavien grading [8] for further analysis, with grades 1 and 2 complications being considered minor and grades 3 and 4 considered major.

All patients were regularly followed up at the outpatient clinic and were prospectively monitored for recurrence. The standard protocol of surveillance included contrast-enhanced computed tomography (CT) scan at one month after resection, followed by liver function test, serum alpha-fetoprotein level check, ultrasonography or CT scan, and chest radiograph every three months. Suspected intrahepatic recurrence was confirmed by hepatic angiography, post-lipiodol CT scan and, if necessary, percutaneous needle biopsy.

The two groups were compared in terms of their baseline clinicopathological features (Table 1). On univariate analysis, there was a significant difference in the preoperative platelet count (*p* = 0.030), international normalized ratio (*p* = 0.035), and resection margin (*p* = 0.023) between the two groups. Furthermore, 16 patients in the PVE group underwent concurrent TACE, but none of the patients in the control group were subjected to this procedure (*p* < 0.001). There was no significant difference in other factors, including maximum tumor size, number of tumor nodules, presence of microsatellite nodules, differentiation, and American Joint Cancer Committee (AJCC) tumor stage. The PVE group had a higher percentage of patients with background cirrhosis, and the control group had a higher percentage of patients with a normal liver. As outcome measures, postoperative complications, disease-free survival, and pattern of tumor recurrence were compared between the two groups.

**Table 1:** Baseline parameters of the portal vein embolization (PVE) group and the control group

	PVE group (n = 34)	Control group (n = 102)	*p* Value^a^
Age, years	57 (27–70)	55 (26–80)	0.883
Males	31 (91%)	78 (76%)	0.082^b^
Status of non-tumorous liver in number of patients			
Non-cirrhotic	2 (6%)	25 (25%)	0.023
Chronic hepatitis	8 (24%)	27 (26%)	0.823
Cirrhotic	24 (70%)	50 (49%)	0.031
Alpha-fetoprotein, ng/ml	105 (2–90,400)	34 (1–530,600)	0.645
Preoperative indocyanine green clearance (%) at 15 min	12.5 (5–28)	10 (3–25)	0.083
Preoperative bilirubin, μmol/l	13 (5–45)	11 (5–145)	0.081
Alanine transaminase, U/l	57 (14–316)	47 (11–393)	0.152
Aspartate aminotransferase, U/l	49 (23–242)	57 (13–223)	0.244
Creatinine, μmol/l	85 (57–133)	85 (44–204)	0.441
Platelet count, 10^9^/ml	179 (126–325)	218 (90–851)	0.030
Albumin, g/l	38.5 (32–46)	41 (20–54)	0.059
International normalized ratio	1 (0.9–1.2)	1 (0.8–2.6)	0.035
Type of resection			1
Right hepatectomy	17 (50%)	51 (50%)	
Extended right hepatectomy	14 (41%)	42 (41%)	
Segmentectomy	3 (9%)	9 (9%)	
Operation duration, min	437 (277–773)	440 (215–883)	0.922
Operative blood loss, l	0.72 (0.2–4.2)	0.9 (0.14–6.2)	0.611
Patients needing transfusion	3 (9%)	11 (11%)	1.000^b^
Hospital stay, days	7 (4–34)	8 (3–61)	0.128
Max. tumor diameter in pathology specimen, cm	7 (3–17)	9 (3–17)	0.177
Resection margin, cm	1 (0.1–3.5)	1 (0.1–6.5)	0.023
Tumor cell differentiation in number of patients			0.682^b^
Well	7 (20%)	17 (17%)	
Moderate	22 (65%)	59 (58%)	
Poor	3 (9%)	17 (17%)	
Not available	2 (6%)	7 (6%)	

Values are expressed as median with range unless indicated otherwise

^a^Mann-Whitney *U*-test, except for ^b^the chi-square test

## Statistics

Statistical comparison between groups was performed using the chi-squared test with the Yates correction (or Fisher’s exact test where appropriate) for nominal data and the Mann-Whitney *U*-test for numerical data. Disease-free survival rates were computed according to the Kaplan-Meier method and compared with the log-rank test. The Cox proportional hazard model was used for multivariate analysis. All analyses were performed with the statistical software SPSS (version 12; SPSS, Chicago, IL). A value of *p* < 0.050 was considered statistically significant.

## Results

### Role of PVE and surgery

In both the resected and non-resected groups of patients, a significant increase of FRLV was noted after PVE. In the non-resected group, the median pre-embolization FRLV was 25% (16–37%) and 29% (18–46%) (*p* = 0.023) after PVE. In the resected group, the pre-embolization FRLV was 23% (12–33.5%), and improved to 34% (20–54%) (*p* = 0.005) after PVE. One patient developed pleural effusion after PVE, which resolved spontaneously. There were no other complications reported after PVE.

**Fig. 1:**
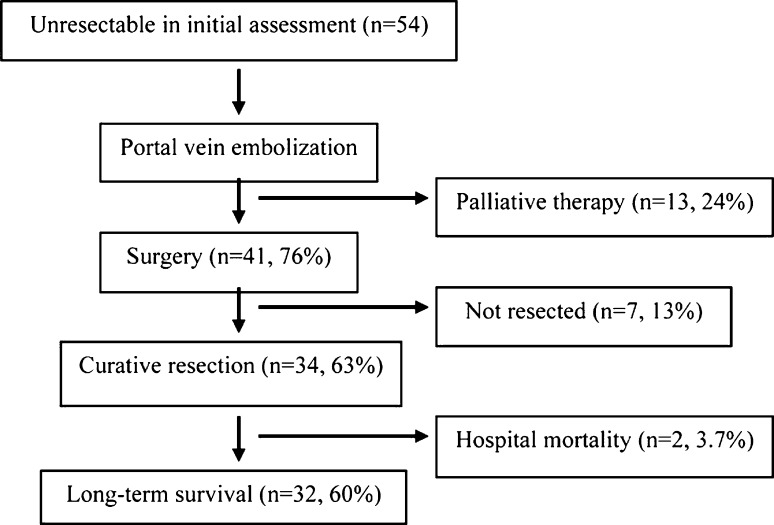
Treatment flowchart of the 54 patients who underwent portal vein embolization (PVE)

Resection was not feasible in 20 (37%) patients. In 10 (18.5%) patients, increase of the FRLV was considered inadequate. In 4 of them, the decision against resection was taken during surgery despite apparently adequate FRLV on the preoperative radiological assessment. In the other 10 patients, surgery was considered not to be feasible due to (1) deteriorated liver function test results in three (5.5%), (2) development of extrahepatic metastasis in three (5.5%), (3) macroscopically grossly cirrhotic liver or extensive varices in two (3.7%), (4) progression of the primary tumor in one (2%), and (5) tumor rupture in one (Fig. 1). Thirty-four (63%) patients underwent curative resection. There were two (3.7%) hospital deaths; one patient died from liver failure and the other developed sepsis and subsequent liver failure. Overall, with the combination of PVE and surgery, 32/54 (60%) of the patients were able to achieve long-term survival (Fig. 1).

### Comparison of the PVE and control groups

Postoperative complications are shown in Table 2. There were two deaths in each group. Minor complications occurred in 10/34 (29%) of PVE patients and 25/102 (24%) of control patients (*p* = 0.651). Major complications were seen in 6/34 (17.6%) of PVE patients and 15/102 (14.7%) of controls (*p* = 0.784).

**Table 2:** Complications in the PVE group and the control group

	PVE group (*n* = 34)	Control group (*n* = 102)	*p* Value^a^
Minor complications	10 (29%)	25 (24%)	0.651
Ascites	7	20	
Wound infection	2	5	
Other	1	–	
Major complications	6 (17.6%)	15 (14.7%)	0.784
Encephalopathy	1	–	
Chest infection	1	2	
Postoperative bleeding	1	1	
Arrhythmia	–	3	
Biliary complication	–	3	
Liver failure	1	3	
Other	–	1	
Mortality	2	2	

^a^Chi-square test

**Fig. 2:**
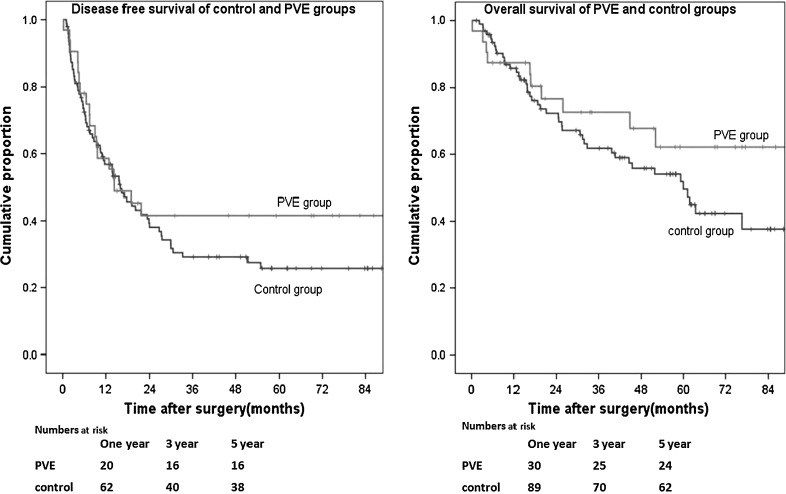
Kaplan-Meier disease-free and overall survival curves of the PVE group (*n* = 34) and the control group (*n* = 102). Disease-free survival, PVE versus control: *p* = 0.335; overall survival, PVE versus control: *p* = 0.221 (log-rank test)

The mean follow-up period in the two groups was 35 months (standard deviation, 25 months). Overall, 14 (41%) patients in the PVE group developed recurrence, compared to 54 (53%) in the control group (*p* = 0.322). Extrahepatic recurrence was detected in 10 (29%) and 41 (40%) cases in the PVE group and the control group, respectively (*p* = 0.310). Intrahepatic recurrence was detected in 10 (29%) of the PVE patients and 47 (46%) of the control patients (*p* = 0.109). There was no difference in the timing of detection of recurrence. Fourteen (41%) of the recurrence cases in the PVE group and 43 (42%) in the control group were detected before one year (*p* = 1.000). The median disease-free survival in the PVE group was 14 months (range: 1.9–94 months), and that in the control group was 13 months (range: 1–88 months). Figure 2 shows the disease-free survival and overall survival of the two groups. The 1-, 3-, and 5-year disease-free survival rates were 57, 29, and 26% respectively, in the control group and 60, 42, and 42%, respectively, in the PVE group (log-rank, *p* = 0.335).

On multivariate Cox regression analysis, venous infiltration (*p* = 0.004; HR = 1.9; 95% CI = 1.2–3), largest tumor diameter (*p* = 0.006; HR = 1.07; 95% CI = 1.02–1.12), and tumor stage (*p* = 0.006; HR = 1.33; 95% CI = 1.08–1.65) were the only individual factors associated with disease-free survival. Portal vein embolization was not a factor affecting disease-free survival (*p* = 0.821; HR = 1.056; 95% CI = 0.65–1.7).

## Discussion

All 54 patients who underwent PVE were not resectable initially due to inadequate FRLV. The combination of PVE and surgery was effective in 60% of these patients. Portal vein embolization was not associated with increased morbidity. Comparison of the PVE group with the controls revealed that the rates of postoperative complications, as well as the pattern of recurrence, were similar between the two groups. There was no difference in disease-free survival between the PVE group and the controls.

Because of concerns for safety and efficacy, PVE was initially limited to normal livers. In a prospective trial, Farges et al. [9] compared the operative outcomes between patients who underwent routine PVE before right hepatectomy and patients who were operated without PVE. Their study showed a clear benefit of PVE in reducing postoperative complications and kinetics of liver function in patients having background chronic liver diseases. No benefit was seen with normal livers. The group advocated routine use of PVE in these patients and further recommended liver regeneration after PVE as a marker of postoperative outcomes. Portal vein embolization has been used for cirrhotic livers with HCC in a number of other centers [10–12], although most of the reported data relate to small numbers of patients. In the present study, a higher proportion of patients in the PVE group had cirrhosis and worsened liver function, and they were expected to have poorer postoperative outcomes. However, the PVE group in fact showed statistically insignificant survival benefit. In this context, our result seems to coincide with that found by Tanaka et al. [6], who reported significantly superior survival in patients with cirrhosis.

Overall, 18.5% of our patients failed to gain adequate increase of FRLV. Twenty-four patients who underwent resection after PVE had cirrhosis. For four other cirrhotic patients who had adequate increase of FRLV, surgery was not performed because of other contraindications. This indicates that 29/44 (66%) of the cirrhotic patients were able to achieve adequate increase of FRLV after PVE. Surgery is known to have the best results in patients with HCC [1]. The outlook for patients with unresectable HCC is bleak; their median survival is reported to be around three months, and their 1-year survival could be as low as 8% [13, 14]. In our series, only one patient developed PVE-related minor complication. Patients in the PVE group tolerated major resection well, and postoperative adverse events were similar in the two groups. As a significant proportion of patients in the PVE group had cirrhosis and poor liver function, these results are even more significant. Similar results have been published in the past by Tanaka et al. [6] and Farges et al. [9] in particular, and both groups recommended routine administration of PVE in patients with injured livers. In view of these reports and our results, PVE should be considered an effective procedure for cirrhotic patients who have stable liver function but are denied resection because of limited FRLV. Routine administration of preoperative PVE in all cirrhotic patients appears to be effective but is beyond the results of the present study.

The risk of progression of primary tumor is a matter of debate. Hepatocellular carcinoma derives its blood supply predominantly from the hepatic artery, and embolization of the portal vein is known to alter the hepatic haemodynamics significantly. Kito et al. [15], using Doppler analysis, demonstrated a significant increase in the arterial flow of the embolized side of the liver without a significant alteration in the contralateral flow. Apart from altered haemodynamics, induction of growth factors that could influence tumor growth has been demonstrated following PVE [16, 17]. Effects of these factors on primary tumor remain the subject of debate.

Most clinical data on tumor kinetics are based on colorectal liver metastasis [18]. These show increased tumor growth during the interval period. In our series, only one patient was found to have primary tumor progression limiting curative resection, whereas three others developed extrahepatic metastasis. In other reported studies on HCC, inoperability due to tumor progression was less than 10% [11, 19]. With these findings taken into account, tumor progression seems a minor clinical problem.

Concurrent use of TACE has been advocated by many authors to control primary tumor and induce liver hypertrophy [20, 21]. The efficacy of this approach remains questionable [22]. We were selective in using TACE in our study group. Almost half (47%) of our patients who had tumors likely to progress or who had a marginal liver volume were offered a combined treatment of PVE and TACE. Only one patient (1/42, 2%) had local tumor progression. Because of the small number of patients in our series, a subgroup analysis of our patients was not performed. However, a recent study from a Korean group compared the outcomes of 71 patients who underwent such combined treatment with the outcomes of 64 patients who had PVE alone [23]. The former group of patients were found to have a more favorable postoperative liver function. They also had better overall survival and disease-free survival. In a smaller but well-planned study on the combined use of PVE and TACE, in addition to better clinical outcomes and survival, pathological specimens showed complete tumor necrosis in 15 of 18 patients [24]. In the present study, the combination of PVE with TACE could have contributed to the comparable outcomes in the PVE group despite the significant proportion of cirrhotic patients. Further TACE was offered to patients who had worse tumors that were likely to become unresectable. Routine administration of PVE combined with TACE might have had a beneficial effect, although this cannot be concluded from this study.

When tumor recurrence was evaluated, we failed to demonstrate any significant difference in disease-free survival or the pattern of recurrence between the PVE and control groups. Further on multivariate analysis, only venous infiltration, tumour stage, and tumor size were shown to be associated with recurrence. To date there are at least three published series focusing on long-term recurrence of HCC after PVE. Tanaka et al. [6] evaluated 30 patients with and without PVE before surgery and showed a better overall survival in patients who had undergone PVE. However, the overall recurrence rate was not statistically different between the two groups. Palavecino et al. [25] reported disease-free survival of 84, 56, and 56% after PVE at 1, 3, and 5 years, respectively, compared to 66, 49, and 49% without PVE. Azoulay et al. [26] reported disease-free survival of 86, 64, and 21% with PVE versus 55, 17, and 17% without PVE at 1, 3, and 5 years, respectively.

The effect of hepatic regeneration on micrometastasis has been studied extensively. De Jong et al. [4] and Mizutani et al. [27] have shown enhanced proliferation of malignant cells in remnant livers after hepatic resection. Some consider that the same growth factors are accountable for hepatic regeneration following PVE, but others contradict this possibility and suggest that a different mechanism may be involved [28, 29]. Vascular permeation is a well-recognized tumor characteristic predicting recurrence. Based on this finding, potential benefits of portal vein obliteration ahead of surgery have been discussed by some authors [30, 31]. Transforming growth factor beta is a polypeptide that suppresses hepatocyte growth and tumor proliferation [32]. Its increased expression has been shown following PVE [33].

It is likely that multiple factors affecting tumor proliferation negatively or positively are brought on by PVE. However, all clinical data indicate that the overall effect of PVE on future recurrence is clinically insignificant. The apparently marginally better recurrence-free survival noticed in clinical studies could possibly be due to the selection of patients during PVE.

In summary, the combination of PVE and surgery was effective in 60% of the patients with initially unresectable HCC. Two thirds of the cirrhotic patients were able to achieve a substantial increase in FRLV. After resection, the patients had rates of morbidity and recurrence-free survival comparable to those of patients who had undergone surgery without PVE. Thus, under the goal of increasing resectability, cirrhosis should not be a limiting factor against PVE for patients with stable liver function.
